# Diagnostic value of combined serological markers in the detection of acute cerebral infarction

**DOI:** 10.1097/MD.0000000000027146

**Published:** 2021-09-10

**Authors:** Xiaowen Zhao, Min Zhao, Baojun Pang, Yingnan Zhu, Jizhu Liu

**Affiliations:** a Department of Clinical Laboratory, Liaocheng People's Hospital, Shandong, China; b Department of Clinical Laboratory, Qilu Hospital of Shandong University, Shandong, China.

**Keywords:** acute cerebral infarction, combination scheme, serological markers

## Abstract

To evaluate the value of the combination schemes of 10 serological markers in the clinical diagnosis of acute cerebral infarction.

The level of total cholesterol, triglycerides, high-density lipoprotein cholesterol (HDL-C), low-density lipoprotein cholesterol, high-sensitivity C-reactive protein, homocysteine (HCY), lipoprotein-related phospholipase A2, ischemia-modified albumin, complement C1q, and lipoprotein a were analyzed in 154 patients with acute ischemic cerebral infarction. The optimized diagnostic combination for acute cerebral infarction was explored by calculating the maximum area under the receiver operating characteristic curves (AUC).

The levels of total cholesterol, triglycerides, low-density lipoprotein cholesterol, high-sensitivity C-reactive protein, HCY, lipoprotein-related phospholipase A2, ischemia-modified albumin, complement C1q, and lipoprotein a were significantly higher in the patient vs the control group. Moreover, the positive rate of HCY reached 89.9%. The analysis of the receiver operating characteristic curve of each index and their combinations showed that the minimum AUC of HDL-C alone was 0.543, while the maximum AUC of HCY was 0.853. A multiple logistic regression analysis indicated that HDL-C was a slightly significant variate in the diagnosis of acute cerebral infarction.

The value of individual serological markers in the diagnosis of acute cerebral infarction was slightly significant, while the combination of the markers significantly improved the efficiency of its diagnosis.

## 1 Introduction

Stroke has become a serious harm to human health and quality of life because of its high morbidity and disability rate.^[1]^ Acute ischemic stroke (AIS) accounts for about 80% of cases of this condition; thus, it is the most common of the various types of stroke.^[1]^ AIS, which is clinically known as acute cerebral infarction, is the most common cerebrovascular disease and causes serious harm to human health.^[2]^ As nearly 10% of these patients die in the initial acute phase of the disease, early diagnosis has been recognized as a key issue in the timely and efficacious treatment of AIS. Moreover, an explicit correlation between acute cerebral infarction and serological markers may provide crucial references for early prevention and treatment. In our study, the level of total cholesterol (TC), triglycerides (TG), high-density lipoprotein cholesterol (HDL-C), low-density lipoprotein cholesterol (LDL-C), high-sensitivity C-reactive protein (hs-CRP), homocysteine (HCY), lipoprotein-related phospholipase A2 (Lp-PLA_2_), ischemia-modified albumin (IMA), complement C1q (C1q), and lipoprotein a [Lp(a)] were analyzed and compared in 154 patients with acute cerebral infarction, to provide a reference for the clinical selection of a more reasonable, economical, and practical combination of serum markers of this condition.

## 2 Patients and methods

### 2.1 Research objects

One hundred fifty-four patients with acute cerebral infarction who were hospitalized at Liaocheng People's Hospital from January 2015 to June 2015 were enrolled in the study. Moreover, all patients were hospitalized within the first 72 hours of the attack. The group was aged 62.4 ± 11.87 years and included 89 males and 65 females who had computed tomography or magnetic resonance imaging (MRI) confirmation of new infarct lesions.^[3]^ According to the *Guidelines for the Diagnosis and Treatment of Acute Ischemic Stroke in China 2010*, the exclusion criteria were: lacunar infarction or recurrent cerebral infarction; cardiogenic pulmonary embolism; severe infection or heart, liver, or kidney disease; tumor or autoimmune diseases; cerebral hemorrhage after infarction; and therapy with hypolipidemic drugs. The maximum infarct size was determined by MRI. The cases were divided into 3 groups according to the maximum diameter of the largest infarct measured by MRI: largest diameter >3 cm: large lesion group (n = 42); largest diameter between 1.5 and 3 cm: medium lesion group (n = 52); largest diameter < 1.5 cm: small lesion group (n = 60). In addition, 42 healthy persons from the database of the physical examination center of Liaocheng People's Hospital (22 males and 20 females; age, 66.05 ± 10.8 years) were used as the control group. The brain MRI of the included patients was abnormal, with a number of detection indicators, including blood pressure, in the normal range. The study was approved by the Liaocheng City People's Hospital Ethics Committee, and all patients or their family members signed the informed consent form for participation. The work described in your article must have been carried out in accordance with The Code of Ethics of the World Medical Association (Declaration of Helsinki).

### 2.2 Reagents and instruments

#### 2.2.1 Detection of TC, TG, HDL-C, LDL-C, and HCY

TC and TG were tested using a terminal colorimetric method; HDL-C was tested by the direct method combined with the selective inhibition method; LDL-C was tested by the direct method combined with the surfactant removal method; and HCY was tested by the enzyme cycle method. The reagents used in the 4 tests were original reagents purchased from Beckman Coulter Inc.

#### 2.2.2 Determination of hs-CRP and Lp-PLA_2_

hs-CRP was tested via the enhanced particle immuno-turbidimetric method, and Lp-PLA_2_ was tested by the continuous monitoring method. The reagents used in this part of study were provided by DiaSys Diagnostics Systems GmbH.

#### 2.2.3 Detection of IMA

IMA was detected by the terminal colorimetric method using reagents provided by Changsha Yikang Technology Development Company.

#### 2.2.4 Detection of complement C1q

C1q was tested by the immune transmission turbidimetric method using reagents purchased from Shanghai Beijia Biochemical Reagent Limited.

#### 2.2.5 Detection of Lp(a)

Lp(a) was detected by the latex enhanced immunoturbidimetry method using reagents provided by F. Hoffmann-La Roche Ltd.

In all samples, detection was performed after calibration and quality control on a Beckman automatic biochemical analyzer (DXC800).

All specimens were collected in the morning under a fasting condition on the second day after hospitalization. The specimens were placed in 3 mL coagulation tubes and centrifuged at 1200 × *g* for 6 min, and the supernatants were transferred to new EP tubes. After labeling with a unified number, the specimens were stored in a refrigerator at −80°C.

#### 2.2.6 Criteria for positive results

After systemic verification, we found that the reference range provided in the reagent manual of each item was applicable to the detection of the population in our region. Therefore, the maximum limit of the reference range in the respective manual was adopted as the positive cutoff value.

### 2.3 Statistical analysis

The data pertaining to TC, HDL-C, LDL-C, Lp-PLA_2_, and C1q levels displayed a normal distribution and are shown as χ ± s, as assessed using the Statistical Product and Service Solutions (SPSS), IBM 17.0 software; moreover, a two-tailed *t* test was adopted for comparisons between the 2 groups. The data of TG, hs-CRP, IMA, and Lp(a) are presented as M (P_25_, P_75_), and the Mann–Whitney *U* test was used to compare the intergroup differences. Multivariate logistic regression was employed to select the risk factors and predict the evaluation index of the diagnostic test; in addition, the results of brain computed tomography or MRI were identified as the gold standard for the diagnosis of acute cerebral infarction. The area under the receiver operating characteristic (ROC) curve (AUC) values, sensitivity, and specificity of the various combinations were analyzed by drawing the ROC curve, and the z test was adopted to evaluate the differences between the various diagnostic combinations and to identify the optimal combinations. Significance was set at *P* < .05.

## 3 Results

### 3.1 Comparison of test items between the acute cerebral infarction group and the control group

The levels of TC, TG, LDL-C, hs-CRP, HCY, Lp-PLA_2_, IMA, C1q, and Lp(a) were higher in the acute cerebral infarction group than they were in the normal group (*P* < .01). Moreover, as the size of the infarction increased, the levels of TC, TG, LDL-C, hs-CRP, HCY, Lp-PLA_2_, IMA, C1q, and Lp(a) increased correspondingly (*P* < .05) (Tables 1 and 2).

**Table 1:** General comparison between the acute ischemic stroke group and the control group.

	Control	Cerebral infarction	*t*/*U*	*P* value
	67.05 ± 10.8	62.41 ± 11.8	0.898	.343
	22/20	89/65	−1.789	.076
Systolic pressure (mm Hg)	122.3 ± 19.12	155.28 ± 23.03	8.981	<.001
Diastolic pressure (mm Hg)	75.9 ± 12.42	91.67 ± 14.72	7.124	<.001
TC (mmol/L)	4.47 ± 0.7	4.79 ± 1.25	4.712	<.001
TG (mmol/L)	0.71 (0.56, 1.83)	1.09 (0.76, 1.64)	−3.907	.000
HDL-C (mmol/L)	1.43 ± 0.27	1.41 ± 0.27	−0.658	.512
LDL-C (mmol/L)	2.17 ± 0.6	3.03 ± 0.8	7.078	<.001
CRP (mg/L)	0.81 (0.42, 1.47)	1.69 (0.7, 4.99)	−3.622	.000
HCY (μmol/L)	10.35 (7.08, 12.05)	11.55 (14.9, 20)	−8.051	.000
Lp-PLA_2_ (U/L)	313.67 ± 104.13	457.4 ± 147.20	7.176	<.001
IMA (U/mL)	69.8 (64.25, 72.7)	75.3 (71.3, 80)	−5.792	.000
C1q (mg/L)	163.68 ± 34.42	181.38 ± 48.86	3.394	.002
Lp(a) (mg/L)	13.05 (3.68, 29.68)	24.2 (11.6, 45.3)	−3.643	.000

**Table 2:** Comparison of each diagnostic item between the acute ischemic stroke group and the control group.

	Cerebral infarction	Large lesions	Medium lesions	Small lesions	Control
Cases	154	42	52	60	42
TC (mmol/L)	4.75 ± 1.2^∗^	4.52 ± 1.44^∗^	4.79 ± 1.31^∗^	4.79 ± 0.98^∗^	3.93 ± 0.9
TG (mmol/L)	1.09 (0.76, 1.64)^∗^	1.16 (0.75, 1.63)^∗^	1.29 (0.89, 1.71)^∗^	1.04 (0.66, 1.64)^∗^	0.71 (0.56, 1.83)
HDL-C (mmol/L)	1.43 ± 0.27	1.36 ± 0.26	1.39 ± 0.26	1.42 ± 0.27	1.3 ± 0.27
LDL-C (mmol/L)	3.07 ± 0.75^∗^	3.14 ± 0.76^∗^	3.02 ± 0.86^∗^	2.96 ± 0.64^∗^	2.17 ± 0.6
CRP (mg/L)	0.81 (0.42, 1.47)^∗^	4.51 (1.72, 8.90)^∗^	1.3 (0.63, 3.2)^∗^	1.05 (0.7, 3.73)^∗^	0.81 (0.42, 1.47)^∗^
HCY (μmol/L)	11.55 (14.9, 20)^∗^	18.4 (14.5, 22.2)^∗^	17.5 (13.4, 21.55)^∗^	16 (12.9, 21.2)^∗^	10.35 (7.08, 12.05)
Lp-PLA_2_	457.4 ± 147.20	468.94 ± 156.39^∗^	466.96 ± 119.79	436.46 ± 150.08	313.67 ± 104.13
IMA	75.3 (71.3, 80)^∗^	76.85 (71.45, 81.82)^∗^	75 (70.98, 79.05)^∗^	73.6 (70.3, 78.1)^∗^	69.8 (64.25, 72.7)
C1q (mg/L)	181.38 ± 48.86^∗^	189.10 ± 55.45^∗^	163.30 ± 45.79	152.65 ± 56.23	163.68 ± 34.42
Lp(a) (nmol/L)	24.2 (11.6, 45.3)^∗^	41.3 (17.3, 50)^∗^	22.6 (6.73, 40.48)^∗^	21.95 (12.83, 39.85)^∗^	13.05 (3.68, 29.68)

### 3.2 Comparison of the positive rate of each item between the acute cerebral infarction group and the control group

**Figure 1:**
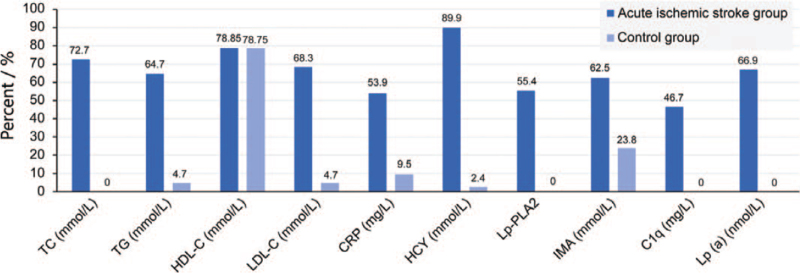
Comparison of the positive rate of each item between the acute ischemic stroke group and the control group. C1q = complement C1q, CRP = C-reactive protein, HCY = homocysteine, HDL-C = high-density lipoprotein cholesterol, IMA = ischemia-modified albumin, LDL-C = low-density lipoprotein cholesterol, Lp(a) = lipoprotein a, Lp-PLA_2_ = lipoprotein-related phospholipase A2, TC = total cholesterol, TG = triglycerides.

The positive rates of TC, TG, LDL-C, hs-CRP, HCY, Lp-PLA_2_, IMA, C1q, and Lp(a) were higher in the acute cerebral infarction group than they were in the control group (*P* < .01), with the positive rate of HCY reaching up to 89.9%. Although the positive rate of HDL-C was up to 78.5% in the case group, this value was not significantly different (*P* > .05) from that of the control group (78.75%) (Fig. 1).

### 3.3 Multivariate logistic regression analysis of the tested items

The multivariate logistic regression analysis showed that LDL-C, hs-CRP, HCY, Lp-PLA_2_, IMA, C1q, and Lp(a) were significant variables for the diagnosis of acute cerebral infarction, whereas TC, TG, and HDL-C were not, with HDL-C being the least significant of the variables (Table 3).

**Table 3:** Multivariate logistic regression analysis of the tested items for the diagnosis of acute cerebral infarction (α input = 0.05, β output = 0.10).

						95% CI	
Variate	Regression coefficient	S.E.	Waldx^2^	*P* value	OR	Lower	Upper
TC	0.459	0.482	0.904	.342	1.582	0.615	4.070
TG	0.476	0.597	0.637	.425	1.610	0.500	5.188
HDL	0.003	0.011	0.060	.806	0.997	0.976	1.019
LDL	0.534	0.945	7.191	.007	12.602	1.978	80.309
CRP	0.551	0.207	7.074	.008	1.735	1.156	2.604
HCY	0.769	0.194	15.789	.000	2.158	1.477	3.155
PLA2	0.015	0.005	10.346	.001	1.015	1.006	1.024
IMA	0.142	0.062	5.158	.023	1.152	1.020	1.302
Clq	2.534	0.945	7.191	.007	12.602	1.978	80.309
Lp(a)	0.068	0.022	9.472	.002	1.070	1.025	1.117
Constant	−41.130	10.140	16.452	.000	0.000		

### 3.4 Comparison of the detection items and various combinations according to area, sensitivity, and specificity under the ROC curve

The analysis of the ROC curve of each item showed that the AUC of HDL-C was the smallest (0.543). Moreover, considering the results reported in the 3 previous subsections together, we demonstrated that HDL-C was of little significance in the diagnosis of acute cerebral infarction. Thus, HDL-C was excluded from the drawing of the ROC curves in the other combinations. In the present study, the combination of TC, TG, and LDL-C, which is denoted as 3 blood lipid items henceforth, was compared with other items in different combinations. The results of these analyses showed that the AUC of the 9-item combination was the largest (AUC, 0.985; sensitivity, 92.8%; specificity, 95.2%) (Table 4). Subsequently, the comparison of the AUC of each item in combination with the 9-item combination accordingly revealed that the AUC-based diagnostic value of HCY alone was the largest (AUC, 0.858; sensitivity, 92.9%; specificity, 60.4%), which was significantly lower than that of the 9-item combination (*P* < .05). The AUC values of the combinations that employed 2, 3, or 4 variates were significantly lower than that of the 9-item AUC (*P* < .05). The combinations of A + B + C + D + E (0.975), A + B + C + D + F (0.969), and A + B + C + D + G (0.98) yielded larger AUC values among the 5-variable groups. Moreover, there were no significant differences in the AUC between these combinations and the 9-item combination (*P* > .05). Finally, the combinations of A + B + C + D + E + F (0.976) and A + B + C + D + E + G (0.985) yielded larger AUC values among the 5-variable groups, with no significant differences between these combinations and the 9-item combinations (*P* > .05).

**Table 4:** Diagnostic value of the combination of 9 different serological markers in acute cerebral infarction.

				95% CI				
Item	Code	AUC	SE	Upper	Lower	Sensibility	Specificity	Youden
Blood lipid	A	0.829	0.034	0.762	0.895	0.667	0.835	0.501
CRP	B	0.685	0.043	0.599	0.77	0.31	0.899	0.209
HCY	C	0.858	0.029	0.8	0.916	0.929	0.604	0.533
Lp-PLA_2_	D	0.798	0.035	0.73	0.866	0.905	0.568	0.473
IMA	E	0.79	0.036	0.719	0.86	0.905	0.64	0.545
C1q	F	0.67	0.041	0.589	0.751	0.738	0.597	0.335
Lp(a)	G	0.686	0.046	0.596	0.775	0.976	0.317	0.293
CH		0.694	0.04	0.615	0.772	0.453	0.952	0.406
TG		0.699	0.45	0.611	0.787	0.734	0.595	0.329
HDL-C		0.543	0.5	0.445	0.64	0.36	0.796	0.145
LDL-C		0.819	0.35	0.75	0.888	0.705	0.81	0.515
	A + B	0.849	0.032	0.787	0.912	0.857	0.698	0.555
	A + C	0.941	0.017	0.907	0.974	0.929	0.813	0.742
	A + D	0.861	0.029	0.804	0.918	0.81	0.777	0.587
	A + E	0.879	0.029	0.822	0.936	0.81	0.813	0.622
	A + F	0.839	0.032	0.776	0.902	0.69	0.849	0.539
	A + G	0.853	0.031	0.792	0.913	0.952	0.64	0.593
	B + C	0.89	0.025	0.841	0.939	0.952	0.669	0.621
	B + D	0.818	0.033	0.755	0.882	0.595	0.871	0.466
	B + E	0.845	0.032	0.781	0.909	0.905	0.748	0.653
	B + F	0.709	0.039	0.632	0.786	0.857	0.583	0.44
	B + G	0.72	0.041	0.64	0.8	0.929	0.439	0.367
	C + D	0.914	0.022	0.872	0.957	0.929	0.763	0.691
	C + E	0.902	0.024	0.855	0.949	0.952	0.719	0.672
	C + F	0.886	0.026	0.835	0.937	0.833	0.791	0.625
	C + G	0.893	0.025	0.844	0.942	0.762	0.856	0.618
	D + E	0.876	0.027	0.823	0.93	0.881	0.741	0.622
	D + F	0.805	0.035	0.737	0.873	0.738	0.748	0.486
	D + G	0.823	0.032	0.76	0.885	0.857	0.705	0.562
	E + F	0.823	0.032	0.761	0.885	0.929	0.655	0.583
	E + G	0.851	0.029	0.794	0.908	0.881	0.691	0.572
	F + G	0.71	0.039	0.634	0.787	0.786	0.597	0.383
	A + B + C	0.954	0.015	0.925	0.982	0.905	0.863	0.768
	A + B + D	0.871	0.027	0.817	0.924	0.81	0.784	0.594
	A + B + E	0.899	0.027	0.846	0.952	0.833	0.827	0.661
	A + B + F	0.853	0.031	0.793	0.914	0.714	0.849	0.563
	A + B + G	0.864	0.029	0.806	0.921	0.905	0.712	0.617
	B + C + D	0.931	0.018	0.896	0.967	0.929	0.784	0.713
	B + C + E	0.929	0.02	0.889	0.968	0.857	0.856	0.713
	B + C + F	0.904	0.023	0.859	0.948	0.905	0.76	0.67
	B + C + G	0.912	0.021	0.871	0.954	1	0.662	0.66
	C + D + E	0.941	0.017	0.908	0.973	0.857	0.892	0.749
	C + D + F	0.918	0.021	0.876	0.959	0.857	O.842	0.698
	C + D + G	0.93	0.019	0.894	0.967	0.952	0.755	0.708
	D + E + F	0.882	0.026	0.83	0.934	0.881	0.755	0.66
	D + E + G	0.893	0.024	0.846	0.94	0.905	0.755	0.66
	E + F + G	0.864	0.027	0.811	0.917	0.833	0.799	0.632
	A + B + C + D	0.968	0.011	0.946	0.99	0.952	0.914	0.866
	A + B + C + E	0.961	0.013	0.935	0.987	0.857	0.935	0.792
	A + B + C + F	0.953	0.015	0.924	0.982	0.881	0.899	0.78
	A + B + C + G	0.962	0.013	0.938	0.987	0.881	0.899	0.78
	B + C + D + E	0.954	0.014	0.926	0.982	0.905	0.885	0.79
	B + C + D + F	0.935	0.018	0.9	0.97	0.929	0.799	0.727
	B + C + D + G	0.947	0.016	0.916	0.977	0.952	0.777	0.753
	C + D + E + F	0.944	0.016	0.913	0.975	0.905	0.871	0.775
	C + D + E + G	0.954	0.015	0.926	0.983	0.905	0.871	0.775
	D + E + F + G	0.896	0.023	0.85	0.942	0.905	0.763	0.667
	A + B + C + D + E	0.975	0.01	0.956	0.994	0.976	0.871	0.847
	A + B + C + D + F	0.969	0.011	0.947	0.99	0.952	0.914	0.866
	A + B + C + D + G	0.98	0.08	0.964	0.996	0.929	0.928	0.857
	B + C + D + E + F	0.955	0.014	0.928	0.983	0.929	0.856	0.785
	B + C + D + E + G	0.965	0.012	0.942	0.988	0.857	0.942	0.8
	C + D + E + F + G	0.956	0.014	0.929	0.984	0.905	0.885	0.79
	A + B + C + D + E + F	0.976	0.1	0.957	0.994	0.892	0.976	0.868
	A + B + C + D + E + G	0.985	0.07	0.972	0.989	0.928	0.952	0.88
	B + C + D + E + F + G	0.966	0.12	0.973	0.998	0.878	0.929	0.806
	A + B + C + D + E + F + G	0.985	0.06	0.973	0.998	0.928	0.952	0.88

## 4 Discussion

The pathogenesis of acute cerebral infarction is a complex process with dynamic changes in relevant indicators and limited therapeutic options. Therefore, the prevention of the various risk factors of this condition is increasingly important. Studies have shown that thrombosis, atherosclerosis (AS), immune factors, and the inflammatory response are involved in the occurrence and development of acute cerebral infarction. Dyslipidemia has been recognized as an important risk factor for AS, as well as a crucial risk factor for ischemic cerebrovascular disease. Numerous reports have confirmed that TC, TG, LDL-C, Lp-PLA_2_, Lp(a), and HCY are involved in the formation of AS. Massot et al^[4]^ found that the activity of Lp-PLA_2_ can be adopted as a high-risk index for identifying other vascular diseases in patients with intracranial atherosclerotic disease. Moreover, studies have shown that an elevated HCY concentration is an isolated risk factor for stroke and cardiovascular disease,^[5,6]^ and that its pathogenesis is associated with AS induced by HCY. Recent studies have also confirmed that AS is a chronic inflammatory process in essence^[7]^; in turn, the high-sensitivity C-reactive protein (hs-CRP), as an acute response protein, is directly involved in the inflammation associated with various cardiovascular and cerebrovascular diseases and has been demonstrated as their most potent predictor and risk factor.^[8]^ Through a variety of mechanisms, hs-CRP can lead to vascular intimal injury, vasospasm, and unstable plaque shedding, thus aggravating the lumen stenosis caused by AS and leading to the occurrence of acute cerebral infarction. In addition, hs-CRP has been recognized as an index for evaluating the severity and prognosis of patients with cerebral infarction.^[9,10]^ An increasing amount of evidence has shown that immune factors and the inflammatory response play a crucial role in the pathophysiology of stroke, and that the activation of the complement system is one of the most important factors in the pathogenesis of brain injury in ischemic cerebrovascular diseases. Consequently, as an important part of the innate immune system in vivo, the complement system is critical in initiating and regulating the process of the inflammatory response.^[11,12]^ As the primary activator of the classical activation pathway, C1q participates in the formation of C3 invertase. Recent studies have confirmed that C1q is positively correlated with the occurrence of acute cerebral infarction.

Although the expression of IMA in ischemic tissues was confirmed by a large number of reports, IMA itself does not have tissue specificity, moreover, it was evidenced in patients with AIS that IMA can lead to definitive reaction to brain injury at various degrees. With the aim of detecting the level of IMA in the serum, Abboud et al^[13]^ conducted an albumin-drilling combined experiment in 60 patients with acute cerebral infarction within 72 hours of its occurrence, and the results showed that the IMA levels were significantly higher in patients suffering from acute cerebral infarction than they were in the healthy control group; this was consistent with the results of the current research.

In this work, 10 serological markers closely related to acute cerebral infarction were analyzed in patients with this condition. The levels of TC, TG, LDL-C, hs-CRP, HCY, Lp-PLA_2_, IMA, C1q, and Lp(a) were significantly higher in the patient group compared with the normal control group (*P* < .01). In addition, as the size of the infarction increased, the levels of TC, TG, LDL-C, hs-CRP, HCY, Lp-PLA_2_, IMA, C1q, and Lp(a) increased correspondingly (*P* < .05), with the positive rate being higher than that of the control group (*P* < .01). Moreover, the positive rate of HCY reached up to 89.9%, which was consistent with the results of previous reports. It is noteworthy that the content of HDL-C in the serum of patients with acute cerebral infarction was not significantly different from that of the normal control group, and no significant change in the content of HDL-C was noted as the size of the infarction increased. There was no significant difference between the case group and the control group regarding the HDL-C positive rate, with HDL-C being the least significant variable, as assessed in the multivariate logistic regression analysis. The drawing of the ROC curve of each item revealed that the AUC of HDL-C was the smallest (AUC, 0.543). Collectively, our results showed that HDL-C was slightly significant in the diagnosis of acute cerebral infarction. In this study, 9 markers in various combinations were used to plot the ROC curve. We found that the AUC was largest for the 9-item combination (0.985). The AUC of HCY alone was the largest (AUC, 0.858; sensitivity, 92.9%; specificity, 60.4%), with this value being significantly lower than that of the 9-item combination (*P* < .05). The comparison of the ROC curves of other combinations with the 9-item combination separately revealed that the combinations of 2, 3, and 4 items failed to achieve an adequate diagnostic level, whereas the AUC increased obviously in the case of the combinations of 5 and 6 items. Moreover, the combinations of A + B + C + D + E (0.975), A + B + C + D + F (0.969), A + B + C + D + G (0.98), A + B + C + D + E + F (0.976), and A + B + C + D + E + G (0.985) were not significantly different; thus, they possessed the same diagnostic value (*P* < .05). In most hospitals, the values of 3 blood lipid items, hs-CRP, and Lp(a) are used in routine examinations. Therefore, the addition of 3 or 4 items can greatly improve the sensitivity and specificity of the diagnosis of acute cerebral infarction, which will not only reduce the financial burden for the patients, but also provide more valuable guidance for clinical examinations. Of note, the neutrophil-to-lymphocyte ratio at admission can accurately predict symptomatic hemorrhagic transformation in patients with AIS undergoing revascularization.^[14,15]^ Moreover, a recent study has demonstrated that the platelet-to-lymphocyte ratio is not only a new marker of atherosclerotic inflammation and a predictive factor in cardiovascular diseases but also an important parameter in diagnosing patients with AIS.^[16]^ These 2 markers can be easily determined clinically and will be explored in a future study.

In summary, this study paved the way for selecting a combination of serum markers with higher sensitivity, stronger specificity, reasonable optimization, as well as economical practicability, thus providing an important reference basis for guiding the clinical diagnosis of acute cerebral infarction. Future analysis should include inflammatory biomarkers, including the neutrophil-to-lymphocyte and platelet-to-lymphocyte ratios, which are highly associated with stroke progression.

## Acknowledgments

Not applicable.

## Author contributions

**Conceptualization:** Jizhu Liu.

**Data curation:** Yingnan Zhu.

**Formal analysis:** Xiaowen Zhao, Min Zhao, Baojun Pang.

**Investigation:** Xiaowen Zhao.

**Methodology:** Xiaowen Zhao.

**Writing – original draft:** Xiaowen Zhao.

**Writing – review & editing:** Xiaowen Zhao.
